# Effectiveness and Safety of the Traditional Chinese Medicine Treatment (*HuoxueHuayu* Therapy) for Malignant Tumors: A Systematic Review and Meta-Analysis

**DOI:** 10.1155/2022/7944063

**Published:** 2022-08-25

**Authors:** Zehui Chen, An Wang, Yue Wei, Yan Zhu, Jing An, Zhiming Li

**Affiliations:** ^1^Institute of Information on Traditional Chinese Medicine, Chinese Academy of Chinese Medical Sciences, Beijing 100700, China; ^2^School of Traditional Chinese Medicine, Beijing University of Chinese Medicine, Beijing 100029, China; ^3^The Third Affiliated Hospital of Beijing University of Chinese Medicine, Beijing 100029, China

## Abstract

**Background:**

A malignant tumor is one of the refractory diseases that threaten human life and health. *HuoxueHuayu* therapy (one of the Traditional Chinese Medicine therapies to promote blood circulation and remove blood stasis) is widely used as an antitumor supplementary method. However, its efficacy and safety are still controversial. Therefore, the objective of this study was to provide evidence-based evidence for *HuoxueHuayu* therapy in the treatment of malignant tumors and confirm its safety and effectiveness.

**Methods:**

A systematic search in 8 electronic databases targeted randomized clinical studies evaluating *HuoxueHuayu* therapy for response evaluation, tumor progression rate, quality of life (QoL), peripheral hemogram, performance status, immunologic function, tumor marker, and blood coagulation function in cancer patients, published from the establishment of the database to December 31, 2020. Risk ratio (RR) was used for counting data, mean difference (MD) or standardized mean difference (SMD) was used for measurement data, and 95% confidence interval (CI) was used as efficacy analysis statistics.

**Results:**

Our search identified 69 studies, evaluating 4402 patients in total. Randomized controlled trials (RCTs) evaluated gastric (*n* = 14), lung (*n* = 18), pancreatic (*n* = 2), colorectal (*n* = 10), liver (*n* = 14), breast (*n* = 2), ovarian (*n* = 2), gallbladder (*n* = 1), esophagus (*n* = 1), and combined (*n* = 14) cancers and hematological malignancies (*n* = 2). The duration of *HuoxueHuayu* therapy ranged from 3 to 48 weeks. Methodological bias was low in 64 studies and high in 5 studies. *HuoxueHuayu* therapy was associated with significant improvement in response evaluation (Response Evaluation Criteria in Solid Tumor (RECIST): RR: 1.44, 95% CI: 1.27 to 1.63, *I*^2^ = 0%, *n* = 33 studies; World Health Organization Criteria in Solid Tumors (WHOCIST): RR: 1.40, 95% CI: 1.23 to 1.59, *I*^2^ = 0%, *n* = 26 studies), recurrence rate (RR: 0.85, 95% CI: 0.72 to 0.99, *I*^2^ = 0%, *n* = 2 studies), quality of life, performance status (MD: 5.60, 95% CI: 5.04 to 6.15, *p* < 0.001), immunologic function (CD3: SMD: 1.23, 95% CI: 0.79 to 1.66, *p* < 0.001; CD4: SMD: 1.25, 95% CI: 0.77 to 1.74, *p* < 0.001; CD4/CD8: SMD: 1.05, 95% CI: 0.69 to 1.42, *p* < 0.001; natural killer cell (NK): SMD: 0.74, 95% CI: 0.32 to 1.15, *p* < 0.001), tumor marker, and blood coagulation function (D-dimer (D-D); fibrinogen (FIB)). In addition, *HuoxueHuayu* therapy could reduce toxicity caused by chemotherapy and radiotherapy without risks of liver and kidney injury or bleeding, although the effect on tumor metastasis was uncertain.

**Conclusions:**

The present update of our systematic review and meta-analyses provided essential evidence for the beneficial effect of *HuoxueHuayu* therapy to show promise in cancer treatment, improving quality of life, addressing cancer-related symptoms, and reducing toxicity in a secure way.

## 1. Introduction

Malignant tumors are the second leading cause of death worldwide [1], and their incidence is increasing year by year [2]. According to the 2020 Cancer Statistics report, there were approximately 19.3 million new cancer cases and 10.0 million cancer deaths worldwide. Furthermore, an estimated 28.4 million new cancer cases may occur in 2040, a 47% increase from the cases in 2020 [3]. Although significant advances in diagnostic screening, surgical resection, and targeted therapies have been made in recent years, the overall situation of malignant tumor treatment is still not optimistic. For instance, patients who are diagnosed at the middle or advanced stages [4, 5] seem to miss the optimum period for surgery [6]. Hence, nonsurgical treatments, such as chemotherapy, radiation therapy, or other treatments (nanomedicines) [7], still play a crucial role in the treatment of middle and advanced malignant tumors.

However, most chemotherapeutic agents are linked to cancer metastasis, the resistance of the drugs, and side effects [8]. In recent years, Chinese herbal medicine, which plays an important role in the treatment of malignant tumors, is believed to have antitumor effects [9]. As a complementary therapy, Chinese herbal medicine enables the reduction of the toxicity and adverse reactions induced by surgery, chemotherapy, and radiotherapy, indicating a synergistic effect with other therapies [10]. Moreover, compared with other drugs, Chinese herbal medicine, which has been widely used for the treatment of malignant tumors in China, is appreciated for its traits of less toxicity and numerous drug targets [11]. According to the theory of Traditional Chinese Medicine (TCM), the formation of a malignant tumor is closely related to blood stasis [12]. Studies have shown that malignant tumors have a high risk of metastasis in blood stasis [13], so herbs to promote blood circulation and remove blood stasis (*HuoxueHuayu* therapy) are often the components of the antitumor formula.

There is no doubt how difficult and necessary it is to prevent the invasion and metastasis of malignant tumors, which are the most essential biological characteristics of malignant tumors [14]. At present, many studies have confirmed that *HuoxueHuayu* therapy allows the reduction of extension or relapse of malignant tumors [13, 15]. However, excessive use of blood-activating drugs may damage the integrity of blood vessels and increase the chance of tumor metastasis [16, 17]. Further, due to the unclear pharmacological mechanism of *HuoxueHuayu* therapy, there is no consensus regarding the clinical application of this treatment, so whether it can prevent or treat malignant tumor metastasis is controversial. Therefore, it is necessary to conduct a systematic evaluation and meta-analysis to provide evidence-based evidence for *HuoxueHuayu* therapy in treating malignant tumors.

## 2. Methods

The review protocol of the previous versions of the systematic review is registered in PROSPERO International Prospective Register of Systematic Reviews (https://www.crd.york.ac.uk/prospero/display_record.php?%20ID=CRD42021247557) and is developed following the guideline of Preferred Reporting Items for Systematic Reviews and Meta-Analyses Protocols (PRISMA-P) [18].

### 2.1. Search Methods

Electronic databases were used, including Embase, PubMed, Elsevier SD, Cochrane Library, Web of Science, Chinese National Knowledge Infrastructure (CNKI), VIP information database, and Wanfang Data Information Site. The retrieval time was from the establishment of the database until December 31, 2020. Search strategies in Embase, for example, were as follows: #1 (“neoplasm”/exp OR neoplasm OR “malignant neoplasm”/exp OR “malignant neoplasm” OR “carcinoma”/exp OR carcinoma OR “leukemia”/exp OR leukemia OR “lymphoma”/exp OR lymphoma) AND ([controlled clinical trial]/lim OR [randomized controlled trial]/lim) AND [1966–2020]/py; #2 (“huoxue” OR “blood stasis syndrome”: ab) AND ([Chinese]/lim OR [English]/lim) AND [humans]/lim AND [clinical study]/lim AND [<1966–2020]/py; #3 #1 AND #2. Hand searches were performed using the bibliographies of all retrieved articles for additional references.

### 2.2. Eligibility Criteria

Inclusion criteria for literature selection were defined using the Population, Intervention, Control, Outcomes, Study Design (PICOS) method (Table 1), including (1) meeting the diagnostic criteria for malignant tumor (excluded metastatic carcinoma), regardless of gender, age or nationality; (2) language only in English and Chinese; (3) randomized controlled trial (RCT). Exclusion criteria were (1) animal studies; (2) retrospective studies; (3) protocols; (4) studies without *HuoxueHuayu* therapy; (5) systematic reviews and meta-analyses; (6) abstract alone; (7) not cancer or irrelevant subject; (8) incomplete data; (9) duplicate articles.

### 2.3. Data Extraction and Management

Two authors (ZHC and AW) independently extracted data from the included studies using Excel. Calibration had been conducted to ensure consistency across reviewers before starting the review. The following information was extracted using a predetermined data form: general information (title, authors, and year of publication); details of the study (aim, random method, inclusion and exclusion criteria, and method of randomization and allocation); study population (age, sex, sample size, number for analysis, type of cancer, and course of cancer); intervention characteristics (type, duration, and blinding of participants and personnel); outcome (main and additional outcomes, time points, method of outcome assessments, blinding of outcome assessment, and adverse effects). Any disagreements were resolved by consensus or consultation with a third review author.

### 2.4. Assessment of Risk of Bias in the Included Studies

Three authors (ZHC, AW, and YW) assessed the risk of bias using the Cochrane Collaboration Risk of Bias Tool [19]. The tool utilizes 10 items to evaluate various sources of bias, including random sequence generation (selection bias), allocation concealment (selection bias), blinding of participants and personnel (performance bias), blinding of outcome assessment (detection bias), incomplete outcome data (attrition bias), selection reporting (reporting bias), and other bias. All evaluated individual domains were endorsed with a “low” (low risk of bias), “high” (high risk of bias), or unclear (insufficient information provided to assess bias), following guideline criteria. The validity of included studies was assessed by the Jadad scale [20], which suggested that 1∼3 were low quality and 4∼7 were high quality. All disagreements among independent bias assessors for a given study item were resolved by team discussion.

### 2.5. Safety Monitoring

Safety monitoring in studies was accessed by explicit reference to formal protocols for systematic monitoring of adverse events, as well as the number and type of intervention-related adverse events reported in the study.

### 2.6. Data Statistical Analysis Methods

We combined more than one trial to estimate pooled intervention effect using the meta-analysis when studies examine the same intervention and outcomes with comparable methods in similar populations. Risk ratio (RR) was used for counting data, mean difference (MD) or standardized mean difference (SMD) was used for measurement data, and 95% confidence interval (CI) was used as efficacy analysis statistics. Heterogeneity was estimated by the *Q*-value together with the *I*^2^ statistic. *p* < 0.1 of *Q*-value or *I*^2^ > 50% indicates statistically significant heterogeneity [21, 22]. The fixed-effect model was used for pooled analysis of homogeneity. The random-effects model was used for data pooled analysis of heterogeneity. Sensitivity analyses were conducted to evaluate the robustness of the pooled results, excluding trials with a high risk of bias. Potential small-study effects, such as publication bias, were explored using Egger's test and funnel plots when at least 10 studies were available, as recommended by the Cochrane Handbook [19]. All analyses were conducted using the Review Manager by the Cochrane Collaboration (version 5.4.1 Copenhagen: The Nordic Cochrane Centre). If a meta-analysis is not possible, we provided a narrative summary of the results from individual studies. Subgroup analyses based on age, sex, type of cancer, duration, type of *HuoxueHuayu* therapy (decoction, injection, capsule, etc.), and type of control group (TCM placebo, chemotherapy, surgery, etc.) were performed to investigate heterogeneity when sufficient data are available.

### 2.7. Evidence Quality Assessment

The overall quality of the evidence for each outcome was rated by the Grading of Recommendations Assessment, Development and Evaluation profiler (GRADEpro) Guideline Development Tool [23]. Two authors (ZC and AW) independently evaluated the overall quality of the body of evidence for each result based on five GRADE criteria, including study limitations, inaccuracies, inconsistencies, indirectness, and publication bias. It is divided into one of four possible ratings (high, medium, low, and very low). Any inconsistencies are resolved by consensus or negotiation with the third review author.

## 3. Results

### 3.1. Literature Search

**Figure 1:**
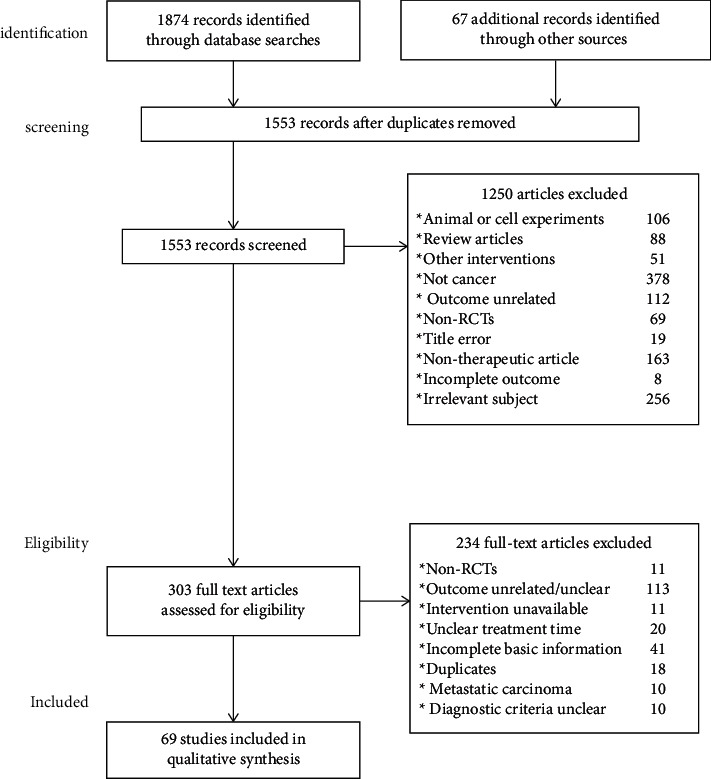
Summary of the flow of our literature search according to the preferred reporting items for systematic reviews and meta-analysis (PRISMA) guidelines. (a) Risk ratio of RECIST for the experimental and control groups. (b) Risk ratio of WHOCIST for the experimental and control groups.

Figure 1 summarizes the flow of the literature search and publication selection process following PRISMA guidelines. The search returned 1874 results from the 8 databases and 67 additional records through manual search. After removing duplicates, there were 1553 unique papers. Titles and abstracts were then reviewed to determine whether papers met inclusion criteria, which resulted in 303 articles for in-depth, full-text review. Full-text reports were then reviewed to further specify whether publications met inclusion criteria, and a final list of 69 publications (RCTs) was established and subjected to the qualitative analysis.

### 3.2. Study Characteristics

The general characteristics of these studies are summarized in Table 2. A total of 4402 cancer patients from the 69 studies (2713 men and 1689 women) were included for systematic review. The majority of studies recruited patients with a single specific cancer type, mostly solid tumor, including 14 gastric cancers [24–37], 18 lung cancers [38–55], 2 pancreatic cancers [56, 57], 10 colorectal cancers [58–67], 14 liver cancers [68–81], 2 breast cancers [82, 83], 2 ovarian cancers [84, 85], 1 gallbladder carcinoma [86], and 1 esophagus cancer [87]. In addition, 2 hematological malignancies [88, 89] and 3 multiples of cancer [90–92] were involved. The number of cases included for each cancer was gastric cancer: 794, lung cancer: 1213, pancreatic cancer: 90, colorectal cancer: 551, liver cancer: 1177, breast cancer: 228, ovarian cancer: 110, gallbladder carcinoma: 40, esophagus cancer: 92, hematological malignancies: 100, prostate cancer: 1, nasopharynx cancer: 1, endometrial carcinoma: 3, and renal cancer: 2.

### 3.3. Intervention and Control Group Characteristics

*HuoxueHuayu* interventions varied in their content, dosage, duration, and intake type. Of the 69 studies, decoction was applied in 60 studies [24–48, 50, 51, 53, 55, 58–62, 64–74, 76–79, 81–89, 91, 92], pill in 2 studies [54, 57], power in 2 studies [49, 90], capsule in 2 studies [63, 80], paste in 1 study [75], injection in 1 study [52], and granules in 1 study [56]. Fifty-seven studies illustrated the *HuoxueHuayu* interventions thoroughly in component and dosage. Lengths of the intervention ranged from 3 to 48 weeks, with an average of 10.6 weeks [24–30, 32–35, 37–46, 48–53, 55, 56, 58,61, 64, 66–79, 82–89, 91].

### 3.4. Risk of Bias Assessment in RCTs

According to the Jadad scale, the quality of RCTs indicated an overall low risk of bias for 64 studies [24–36, 38–53, 55–72, 75–86, 88–92] and overall high risk of bias for 5 studies [37, 54, 73, 74, 87] (Table 3). Among 69 RCTs, 34 studies (49.2%) reported a specific random sequence [26, 28, 30, 33, 38–42, 44, 46 –48, 50, 51, 53, 56, 58, 59, 62, 64, 68–70, 75, 76, 79, 80, 84, 86, 88–90, 92], but only 4 studies (5.8%) [28, 41, 47, 78] mentioned the allocation concealment process. Blinding of participants was mentioned in 2 studies [28, 41], and none of the studies reported blinding of outcome assessment among all included studies. Only one study had incomplete outcome data that was unable to ensure its impact on the whole result of the research [38]. All studies reported the timing of outcome assessments without other bias.

### 3.5. Main Outcomes

#### 3.5.1. Response Evaluation

Response evaluation of cancer was assessed in 59 studies, using the following criteria: Response Evaluation Criteria in Solid Tumors (RECIST, 33 studies) [24–26, 29–33, 39, 41–44, 46, 49, 50, 52, 53, 58–62, 64, 68, 71, 75, 82, 84, 85, 87, 90, 92] and World Health Organization Criteria in Solid Tumors (WHOCIST, 26 studies) [27, 34–38, 45, 51, 54, 56, 57, 63, 65–67, 69, 70, 72–74, 76, 77, 79–81, 86].

**Figure 2:**
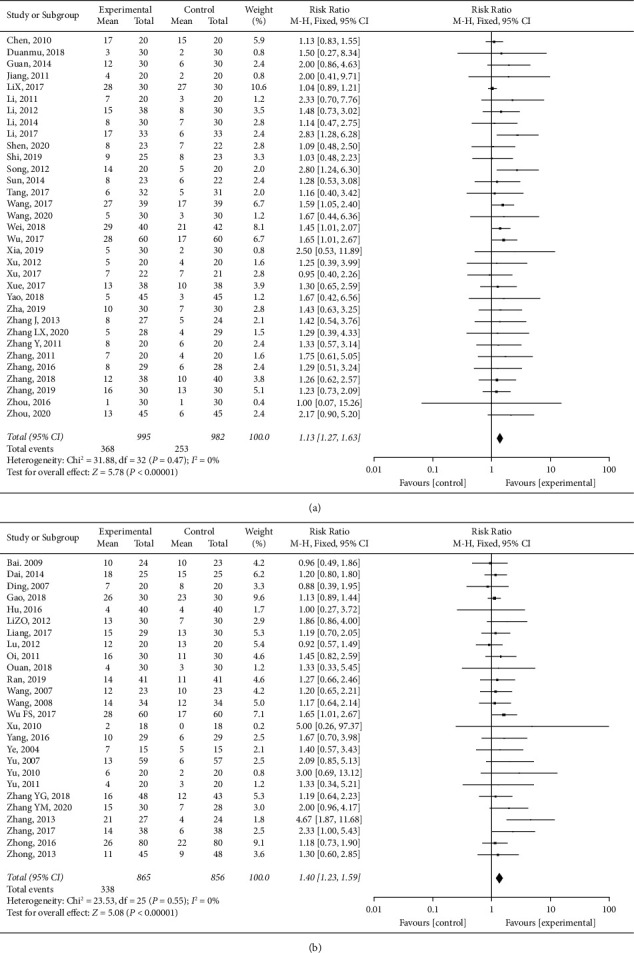
Response evaluation of *HuoxueHuayu* therapy for malignant tumor.

The overall effect size based on a fixed-effect model indicated a beneficial effect of *HuoxueHuayu* therapy on response evaluation in cancer patients (RECIST: RR: 1.44, 95% CI 1.27 to 1.63, *I*^2^ = 0%, *n* = 33studies; WHOCIST: RR: 1.40, 95% CI 1.23 to 1.59, *I*^2^ = 0%, *n* = 26 studies). The corresponding forest plots are given in Figure 2.

#### 3.5.2. Tumor Progression Rate

The tumor progression rate of cancer was assessed via metastasis rate [40] and recurrence rate [40, 78]. The results showed that *HuoxueHuayu* therapy was a protective factor in decreasing the recurrence rate (RR: 0.85, 95% CI 0.72 to 0.99, *I*^2^ = 0%, *n* = 2 studies). Only one study emphasized the effect of *HuoxueHuayu* therapy on tumor metastasis (RR: 0.83, 95% CI 0.40 to 1.73), and the result was equivocal due to inadequate evidence.

#### 3.5.3. Quality of Life

Quality of life was assessed in 22 studies using the following criteria: the European Organization for Research and Treatment of Cancer-Quality of Life Questionnaire (EORTC QLQ-C30, 14 studies) [25, 26, 29–31, 33, 34, 48, 53, 58, 68, 84, 85, 91], Functional Assessment of Cancer Therapy-Colorectal (FACT-C, 2 studies) [60, 62], the European Organization for Research and Treatment of Cancer-Quality of Life Questionnaire for Breast Cancer (EORTC QLQ-BR23, 1 study) [83], and a questionnaire designed by researchers based on international criteria (5 studies) [28, 32, 64, 65, 77]. In terms of the overall score of the quality of life questionnaire, 18 studies of which outcomes demonstrated five conditions, including quality of life promotion in the experimental group combined with no change in the control group [28, 30, 31, 33, 34, 48, 53, 60, 62], quality of life promotion in the experimental group combined with the reduction in the control group [32, 44, 83, 91], quality of life promotion in the experimental and control group [25, 65, 68], no change of quality of life in the experimental group combined with the reduction in the control group [26], and quality of life reduction in the experimental and the control group [77], reflected the significant difference between the experimental group and the control group. Four studies showed similar efficiency in the experimental group and the control group, and two of them manifested a significant quality of life promotion after *HuoxueHuayu* therapy [29, 58]. In general, *HuoxueHuayu* therapy showed a positive trend in improving the quality of life or slowing down the degradation of the quality of life.

#### 3.5.4. Peripheral Hemogram

Two studies indicated the significant efficiency of *HuoxueHuayu* therapy in reducing the degree of WBC decline (*p* < 0.05) [65, 88] and improving the hemorheological parameters (plasma viscosity, whole blood reduced high shear viscosity, and low shear viscosity reduction of whole blood) for hematological malignancies (*p* < 0.05) [89]. There were no significant differences between the experimental group and the control group in APTT, D-D, or FIB (*p* < 0.05) except PT (*p* < 0.05), which meant that *HuoxueHuayu* therapy could improve blood hypercoagulability in patients with hematologic malignancies without increasing the risk of bleeding.

### 3.6. Additional Outcomes

#### 3.6.1. Performance Status

**Figure 3:**
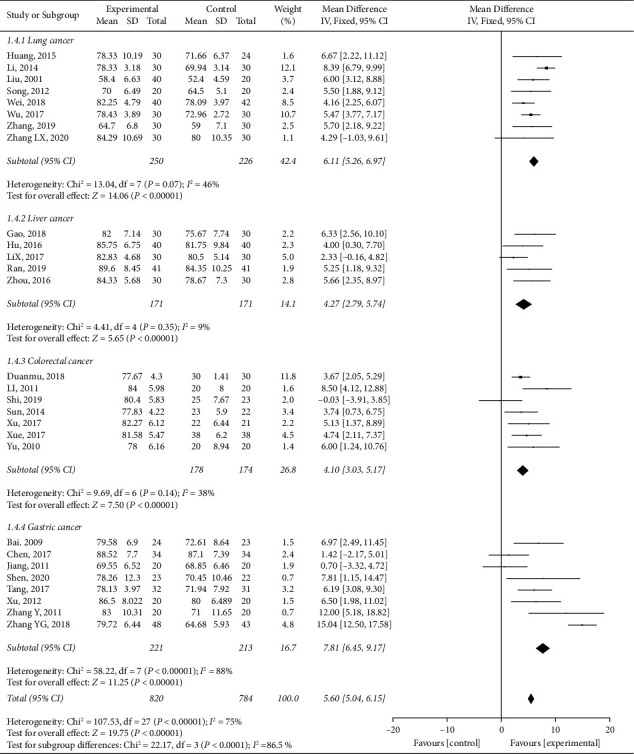
Performance status of *HuoxueHuayu* therapy for malignant tumor (KPS score). (a) CD3 level of *HuoxueHuayu* therapy for malignant tumor. (b) CD4 level of *HuoxueHuayu* therapy for malignant tumor. (c) CD8 level of HuoxueHuayu therapy for malignant tumor. (d) CD4/CD8 level of *HuoxueHuayu* therapy for malignant tumor. (e) NK level of *HuoxueHuayu* therapy for malignant tumor.

Performance status was evaluated in 32 studies by the Karnofsky score (KPS). Data from 28 RCTs were pooled for analysis. The *Q*-value (*p* < 0.001) suggests substantial heterogeneity with an *I*^2^ of 75%. The overall effect size based on a fixed-effects model indicated a statistically significant trend in favor of *HuoxueHuayu* therapy on cancer-related performance status (MD: 5.60, 95% CI: 5.04 to 6.15, *p* < 0.001). Subgroup meta-analyses according to cancer type, including lung cancer (MD: 6.11, 95% CI: 5.26 to 6.97, *p* < 0.001, *I*^2^ = 46%) [39, 41–43, 47, 49, 53, 55], liver cancer (MD: 4.27, 95% CI: 2.79 to 5.74, *p* < 0.001, *I*^2^ = 9%) [69–71, 75, 77], colorectal cancer (MD: 4.10, 95% CI: 3.03 to 5.17, *p* < 0.001, *I*^2^ = 38%) [58–62, 64, 65], and gastric cancer (MD: 7.81, 95% CI: 6.45 to 9.17, *p* < 0.001, *I*^2^ = 88%) [24, 28–31, 33, 34, 37], showed similar trends. The KPS of esophagus cancer [87], ovarian cancer [84], and multiple types of cancer [90, 91] showed that *HuoxueHuayu* therapy could significantly improve the performance status of malignant tumor patients compared to conventional treatment (Figure 3).

#### 3.6.2. Survival Condition

Five studies introduced survival access using survival rate, median survival time, and progression-free survival. The results revealed that *HuoxueHuayu* therapy was a protective factor in increasing median survival time (*p* < 0.05) [52, 76, 78] and survival rate (*p* < 0.05) [55, 76, 78]. However, there was no significant difference between the experimental group and the control group in progression-free survival (*p* > 0.05) [47] due to the scarce studies.

#### 3.6.3. Immunologic Function

**Figure 4:**
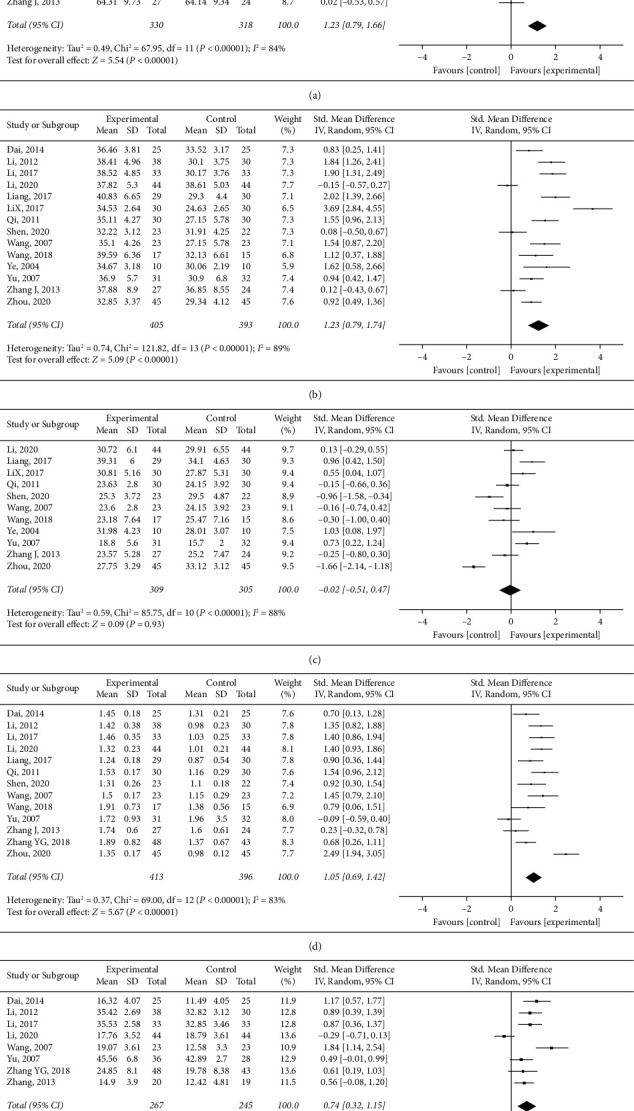
Immunologic function of *HuoxueHuayu* therapy for malignant tumor (CD3, CD4, CD8, CD4/CD8, and NK cell level). (a) APTT of *HuoxueHuayu* therapy for malignant tumor. (b) PT of *HuoxueHuayu* therapy for malignant tumor.

Immunologic function was valued by the level of CD3, CD4, CD8, CD4/CD8, and NK cells. The *Q*-value (*p* < 0.001) suggested substantial heterogeneity with *I*^2^ of 84% for CD3, 89% for CD4, 88% for CD8, 83% for CD4/CD8, and 80% for NK. All meta-analysis with a random-effects model on the 17 studies unveiled a beneficial effect of *HuoxueHuayu* therapy on CD3 (SMD: 1.23, 95% CI: 0.79 to 1.66, *p* < 0.001) [24, 35, 36, 40, 44, 52, 54, 56, 73, 80, 83, 92], CD4 (SMD: 1.25, 95% CI: 0.77 to 1.74, *p* < 0.001) [24, 35, 36, 40, 44, 52, 54, 56, 71, 73, 80, 82, 83, 92], CD4/CD8 (SMD: 1.05, 95% CI: 0.69 to 1.42, *p* < 0.001) [24, 35, 37, 40, 44, 52, 54, 56, 73, 80, 82, 83, 92], and NK cell levels (SMD: 0.74, 95% CI: 0.32 to 1.15, *p* < 0.001) [35, 37, 38, 40, 44, 52, 56, 80], while a statistically nonsignificant trend in favor of CD8 suggested that the effect of *HuoxueHuayu* therapy was unsure because of the limited evidence (SMD: −0.02, 95% CI: −0.51 to 0.47, *p*=0.93) [24, 35, 36, 40, 54, 71, 73, 80, 82, 83, 92] (Figure 4).

#### 3.6.4. Tumor Marker

Thirty studies with 1961 patients reported the level of tumor markers including CA125 (8 studies), CEA (20 studies), CA153 (2 studies), AFP (11 studies), AFU (1 study), CA199 (14 studies), CYFR21-1 (2 studies), VEGF (2 studies), CA50 (1 study), and CA724 (1 study). Fourteen studies with 759 patients showed no statistically significant decline in tumor markers of *HuoxueHuayu* therapy compared with conventional treatment [25, 30–33, 53, 58, 59, 62, 65, 71, 78, 84, 91], and 6 of them suggested that neither *HuoxueHuayu* therapy nor conventional treatment could significantly lower the tumor marker level (*p* > 0.05) [30, 33, 53, 58, 62, 65], but 4 of them revealed a positive effect in decreasing tumor markers of *HuoxueHuayu* therapy (*p* < 0.05) [25, 31, 59, 84]. Fifteen studies with 1112 patients indicated that the effect of *HuoxueHuayu* therapy in decreasing tumor markers was better than conventional treatment (*p* < 0.05) [24, 36, 39, 40, 50, 61, 68–70, 72, 74, 77, 79, 80, 83], typically in the level decline of CEA (7 studies) [36, 39, 40, 50, 61, 68, 83] and AFP (8 studies) [68–70, 72, 74, 77, 79, 80]. Overall, whether the *HuoxueHuayu* therapy could efficiently reduce the tumor marker level was still controversial due to poor evidence.

#### 3.6.5. Blood Coagulation Function

**Figure 5:**
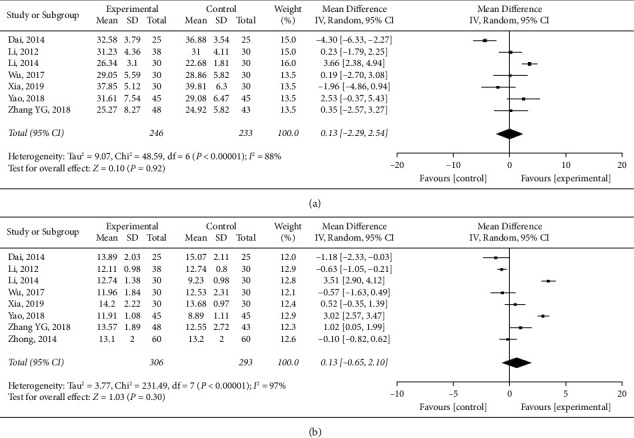
Blood coagulation function of *HuoxueHuayu* therapy for malignant tumor (APTT and PT).

Blood coagulation function was assessed in 14 studies using PT (8 studies), PLT (5 studies), APTT (7 studies), FIB (8 studies), D-D (11 studies), TT (1 study), INR (1 study), and PTA (1 study). Twelve studies implied that *HuoxueHuayu* therapy had a trend of the intervention of blood hypercoagulability compared with the conventional treatment (*p* < 0.05), especially in improving the level of D-D (*p* < 0.05) [29, 43, 49, 52, 56, 57, 90, 92] and FIB (*p* < 0.05) [26, 37, 43, 48, 49, 52, 90, 92]. Meta-analysis based on a random-effects model showed that there were no statistically significant differences between the experimental group and the control group of APTT (MD: 0.13, 95% CI: −2.29 to 2.754, *p*=0.92, *I*^2^ = 88%) [26, 37, 43, 49, 52, 56, 90] and PT (MD: 0.72, 95% CI: −0.65 to 2.10, *p*=0.30, *I*^2^ = 97%) [26, 37, 43, 49, 52, 56, 78, 90], meaning the unobvious effect of *HuoxueHuayu* therapy on ATPP and PT. One study reported improvement in INR and PTA [69] of *HuoxueHuayu* therapy, yet one study revealed a nonsignificant trend in favor of TT [37]. Four studies indicated positive effects of *HuoxueHuayu* therapy on PLT (*p* < 0.05) [48, 49, 56, 90] except one study [78]. Since the measurement units of D-D, FIB, and PLT were not identical, we just described the results instead of data synthesis (Figure 5).

### 3.7. Reports of Safety and Adverse Events

Sixty studies mention the safety and adverse events of the treatment. Except for six [27, 45, 48, 81, 83, 89] studies without any adverse events, 54 studies revealed that the toxicity was caused by chemotherapy and radiotherapy, including myelosuppression (anemia, leucopenia, and thrombocytopenia), gastrointestinal reactions (nausea, emesis, and diarrhea), alopecia, pain, hepatic injury, renal injury, fatigue, hot flash, hypertension, sleepiness, hand-foot syndrome, radioactive dermatitis, and radiation pneumonitis. Nine studies showed that the toxicity of experimental group and control group was no statistically significant differences (*p* > 0.05), while 45 studies reflected the positive effect of *HuoxueHuayu* therapy in reducing the toxicity, mainly in promoting leucopenia (17 studies) [26, 29, 32, 35, 37, 38, 46, 51, 52, 54, 57, 59, 63, 66, 74, 76, 88], nausea and emesis (31 studies) [24, 30–32, 35, 36, 38, 41, 43, 44, 46, 47, 50–52, 54–59, 63–66, 70, 74, 82, 84, 86, 88], diarrhea (17 studies) [29–31, 36, 38, 40, 41, 43, 44, 47, 51, 56–58, 64, 66, 75], thrombocytopenia (6 studies) [32, 36, 38, 46, 86, 88], and hepatic injury (5 studies) [25, 32, 56, 72, 80] (*p* < 0.05). All the studies reported no severe toxicity of anticancer drugs at stage III or IV based on the WHO standard.

### 3.8. Overall Quality of Evidence according to Outcome Measures

Compared *HuoxueHuayu* therapy with conventional treatment, the overall quality of evidence according to outcome measures was high in 2 results (RECIST; WHOCIST), moderate in 1 result (KPS score), low in 3 results (recurrence rate, CD4/CD8, and CD3), and very low in 5 results (APTT, PT, CD4, and CD8). The results of GRADE assessments are presented in Table 4.

## 4. Discussion

Based on the TCM theory, *HuoxueHuayu* therapy is prevalently applied for malignant tumor treatment. It is often combined with chemotherapy, radiotherapy, and surgery, and its efficacy has been testified in a large sum of clinical trials. However, the safety of *HuoxueHuayu* therapy has been controversial, as it seems to promote tumor metastasis and thus makes the cancer therapy uncertain to be recognized in clinics. Therefore, we summarized the clinical trials, mainly in RCTs, to illustrate the effect and safety of *HuoxueHuayu* therapy in this review. To assess the efficiency of *HuoxueHuayu* therapy, we utilized four main outcomes and five additional outcomes to explain the effects from various aspects. As the main outcome, response evaluation of cancer distinctly reflected the improvement of cancer, typically in the solid tumor before and after treatment, which had been widely accepted by therapeutic evaluation of malignant tumors using RECIST and WHOCIST. We employed metastasis rate and recurrence rate as tumor progression rate to evaluate the safety of *HuoxueHuayu* therapy. In addition, as many researchers were concerned, we focused on the quality of life of patients with cancer in terms of the subjective feeling of patients to estimate the effect of treatment, which was an essential approach in therapeutic evaluation. Some cancers like hematological malignancies could hardly be measured by RECIST and WHOCIST; hence, peripheral hemogram, including WBC, RBC, LYM, PT, PLT, APTT, FDP, and D-D, was used to describe the changing in cancer treatment. Besides that, we added some other outcomes to thoroughly demonstrate the influence of *HuoxueHuayu* therapy from the perspective of immunologic function, activity performance of patients (KPS score), tumor indicators (tumor marker), and the prognosis of cancer (survival condition). Meanwhile, we introduced the results of blood coagulation function as an additional outcome since it was necessary to clarify if *HuoxueHuayu* therapy could increase the risk of bleeding, which was related to safety.

Our finding revealed that *HuoxueHuayu* therapy minimized the size of tumor combined with conventional treatment, which was better than chemotherapy or radiotherapy alone, meaning that *HuoxueHuayu* therapy might reinforce the effect of conventional treatments. Nearly all cancer patients suffer from physical and psychological trauma caused by tumors and the toxicity of drugs. We found that the intervention of *HuoxueHuayu* therapy could improve the quality of life compared to pretreatment or slow down the deterioration of life quality after treatment when adverse effects were inevitable. In particular, *HuoxueHuayu* therapy might significantly relieve fatigue and increase appetite [84], which was consistent with the results of the KPS score and adverse events. Some researchers regard the KPS score as an assessment of life quality, while the KPS score reflects the behavioral capacity of patients without psychological evaluation, so we extracted the KPS score from a life quality assessment to illustrate, respectively. *HuoxueHuayu* therapy manifested a beneficial trend of improving performance status in various tumor types, indicating that patients with cancer were likely to have a better prognosis [93]. Just as the results of survival condition reflected, *HuoxueHuayu* therapy improved prognosis by increasing median survival time [52, 76, 78] and survival rate [55, 76, 78]. Although there are limited relevant studies, we could not neglect the effect of *HuoxueHuayu* therapy in prolonging patient survival.

The activity of the immune system concerning cancer is an important factor in the prognosis of patients with cancer. Many cytotoxic chemotherapy drugs have been shown to induce immunogenic cell death [94], while others reduce the number and function of immunosuppressive cells in the tumor microenvironment [95]. Chemotherapeutic drugs enhance *T* lymphocytes, including CD4, CD3 [96], and NK cells in patients with malignant tumors, which are often in the inactive or inhibited state [97]. Our findings showed that *HuoxueHuayu* therapy might have immunomodulatory activity by elevating the *T* cells and NK cells to suppress tumor growth, acting as potential tumor immunotherapy. The content of CD8 and CD4 maintains a dynamic balance in the healthy state of the body to ensure the normal development of the human immunologic function, but the immune function will be greatly inhibited with high content of CD8, typically in cancer tissues. According to the result, *HuoxueHuayu* therapy on CD8 levels was not clear, so more evidence would be needed to verify the effect of drug action.

A tumor marker is a substance produced by or associated with a tumor, found in the blood in concentrations higher than in malignancy-free subjects. It is believed that, as potentially good indicators, tumor markers can reflect the severity of diseases and predict tumor recurrence and metastasis to a certain extent. Therefore, the decrease in tumor markers could be regarded as a favorable prognostic trend. We discovered that *HuoxueHuayu* therapy had an obvious advantage in reducing CEA and AFP, but the tumor markers CEA and AFP can be found in various cancers instead of unique cancer, so they lack tumor specificty. These results were consistent with the improvement of tumor size and life expectancy, meaning that the decline in tumor markers was due to improvements in cancer by *HuoxueHuayu* therapy.

In this review, we focused on the peripheral hemogram changes in hematologic malignancies and the blood coagulation function. Based on our research, *HuoxueHuayu* therapy is inclined to regulate the WBC, suggesting that it might have more advantages in preventing infection than conventional treatment. Studies have shown that blood hypercoagulability, which can affect the biological behavior of tumor cells and thus promote tumor development and metastasis, is closely related to the occurrence, development, and prognosis of malignant tumors [98, 99]. *HuoxueHuayu* therapy had a trend of the intervention of blood hypercoagulability typically in D-D, FIB, PLT, and hemorheological parameters, without bleeding tendency. Some procoagulant substances can promote tumor cell proliferation, angiogenesis, and distant metastasis [100]. Our results showed that *HuoxueHuayu* therapy was a protective factor in decreasing the recurrence rate, but the result on tumor metastasis was equivocal due to the inadequate evidence, which indicated that the effect of *HuoxueHuayu* therapy on hypercoagulability and tumor metastasis was not completely synchronized.

TCM considers malignant tumor as a systemic disease, not only the local lesion but also the systemic functions affected by cancer, so the therapy concept is holistic and combined with local treatment, which is the advantage of TCM in the treatment of malignant tumors. Based on the TCM theory, a malignant tumor is closely related to blood stasis, meaning that exposure to blood stasis and *qi* stagnation for a long time can lead to a “cancerous toxin” which causes cancer. Blood stasis and “cancerous toxin” are homologous, and blood stasis already exists in the initial stage of the tumor, which is conducive to the formation of cancerous toxin to promote tumor metastasis [101, 102]. Blood stasis is a key etiology factor in the genesis, invasion, and metastasis of tumors, which provides a reliable basis for the treatment of cancer by *HuoxueHuayu* therapy. However, blood stasis usually influences the development of cancer by combining other pathogenic factors such as *qi* deficiency, heat, and toxins rather than only by itself, which makes the therapy more compound to concern all the aspects instead of a unique method. In our review, we found that abundant researchers used *HuoxueHuayu* therapy combined with other methods, including tonifying *qi*, detoxication, and clearing heat, to implement the integral efficacy.

In terms of the mechanism, *HuoxueHuayu* prescription was confirmed to refrain tumor cell proliferation [32, 44, 61, 62], adjust the equilibrium of MMP-2/TIMP-2 [44], abnormal miRNA expression such as miRNA-21, miRNA-143, miRNA-145, and miRNA-146a [61, 62], VEGF, and Ki-67 [32] to refrain tumor, and play a role in antitumor angiogenesis effect. Besides that, multiple systematic reviews have summarized the potential mechanisms of *HuoxueHuayu* therapy as follows [103–105]: (1) regulating related genes; (2) inhibiting tumor cell proliferation and metastasis; (3) promoting tumor cell apoptosis; (4) inhibiting angiogenesis; (5) modulating immune function; (6) reversing drug resistance of cancer cells. Although the mechanism of reconstitution and regulation of some immune cells and immunosuppressive cells are roughly the same between *HuoxueHuayu* therapy and *HuoxueHuayu* therapy combined with tonifying *qi*, in the intensity of regulation, the later one is superior to the former [105]. We should emphasize that *HuoxueHuayu* therapy achieves therapeutic effects through the action of multiple herbs rather than a single herb or an ingredient of herb, although some herbs have been reported to tend to promote tumor metastasis [15, 16, 106].

According to this study, we recommended the use of *HuoxueHuayu* therapy in addition to conventional treatment because it could relieve the side effect of chemotherapy and radiotherapy. The adverse reaction of the digestive system (nausea, emesis, and diarrhea) and immunosuppression (leucopenia) was prone to be the prominent target of *HuoxueHuayu* therapy, which might be related to its effect on improving gastric motility and strengthening gastrointestinal and immune function [107–109]. It was positive to see that *HuoxueHuayu* therapy improved blood circulation and reduced blood viscosity without any bleeding cases reported, indicating its reliable safety and accessibility. Meanwhile, *HuoxueHuayu* therapy could lower the accidence of hepatic injury without any reported renal injury, confirming that it not only avoided enhancing the toxicity but also reduced the risk of adverse events by combining it with conventional treatment.

### 4.1. Limitations and Suggestions for Future Research

We confronted some limitations based on published literature so that our conclusion might be conserved. First, the included papers were limited to English and Chinese, especially Chinese that accounted for the majority, which would lead to publication bias. Second, because of the deficient research, we could not provide a confirmed conclusion on some outcomes such as the intervention of *HuoxueHuayu* therapy on tumor metastasis, so it is still hard to recognize its stimulation or inhibition. We are still prone to believe that *HuoxueHuayu* therapy can suppress tumor metastasis and prolong the survival time because, among a robust and growing body of evidence, the effect of *HuoxueHuayu* therapy on antitumor metastasis has been confirmed by animal and in vitro experiments [110–112]. Third, due to the inconsistent measurement and heterogeneity of outcomes reported in these studies, the meta-analysis was by necessity limited to the most common outcome measures without full coverage quantification of results. Fourth, the pooling of studies that were done for meta-analysis did not consider the diversity and complexity of patients with tumors across studies and thus limited the inferences that could be drawn regarding the benefits of *HuoxueHuayu* therapy to specific cancer types. Lastly, the quality of evidence according to outcome measures is concentrated in low or very low due to inconsistency, imprecision, and publication bias, so rigorously designed studies will need to be undertaken to confirm the conclusions. In terms of demographic features, future reviews should further stratify results based on the type of cancer, age, or other groupings. Meanwhile, whether the forms or the dose of herbs contributing to *HuoxueHuayu* therapy will influence the curative effect or not should be studied deeply.

## 5. Conclusion

In conclusion, our findings would supplement current effective therapies by providing essential evidence for the beneficial effect of *HuoxueHuayu* therapy. We systematically summarized the effects of *HuoxueHuayu* therapy on the improvement of cancer, recurrence rate, quality of life, performance status, immunologic function, blood status, and immunity of patients with malignant tumors to confirm that it showed promise in cancer treatment, improving quality of life, addressing cancer-related symptoms, and securely reducing toxicity. Besides, *HuoxueHuayu* therapy demonstrated its therapeutic effect on various malignant tumors through the compatibility of TCM rather than merely depending on a few herbs. In further efforts to strengthen uniformity in clinical trial reporting, more extensive and methodologically sound trials with more extended follow-up periods are needed.

## Figures and Tables

**Table 1:** Population, intervention, control, outcomes, and study design (PICOS) inclusion criteria.

Item	Content
Population	Patients with cancer (excluding metastatic carcinoma)
Intervention	*HuoxueHuayu* therapy alone or combined with the intervention of control group
Control	Use conventional treatment only with Western medicine
Outcomes	
Main: response evaluation, QoL, tumor progression rate, and peripheral hemogram (only for hematological malignancies)	Tumor progression rate included recurrence rate and metastasis rate. Peripheral hemogram included WBC, RBC, LYM, PT, PLT, APTT, FDP, and D-D
Additional: performance status, survival condition, immunologic function, tumor marker, and blood coagulation function	Blood coagulation functions included PT, PLT, APTT, FDP, D-D, and FIB. Immunologic function included CD3, CD4, CD8, CD4/CD8, and NK cell
Study design	RCT

APTT, activated partial thromboplastin time; D-D, D-dimer; FDP, fibrinogen degradation products; FIB, fibrinogen; KPS, Karnofsky score; LYM, lymphocyte; NK, natural killer; PLT, platelets; PT, prothrombin time; QoL, quality of life; RBC, red blood cell; WBC, white blood cell.

**Table 2:** General study characteristics of included studies (RCTs).

Study	Type of cancer	Intervention		Number (male/female)		Age		Duration (week)	Outcome measures
		Experimental	Control	Experimental	Control	Experimental	Control		
Qi, 2011	LC	Chemotherapy and TCM	Chemotherapy	20/10	19/11	59.26 ± 9.42	57.39 ± 8.38	6	WHOCIST and immunologic function
Zhou, 2020	BC	BSC and TCM	BSC	45 (F)	45 (F)	48.2 ± 5.9	47.6 ± 5.4	12	RECIST, tumor marker, and immunologic function
Zhang, 2013	GBC	Chemotherapy and TCM	Chemotherapy	7/13	6/14	67.50 ± 9.24	68.40 ± 6.98	6	WHOCIST and immunologic function
Huang, 2015	LC	Chemotherapy and TCM	Chemotherapy	22/8	16/8	60.36 ± 8.58	57.62 ± 10.65	8	Progression-free survival and KPS
Zhou, 2013	LC	Chemotherapy and TCM	Chemotherapy	25/20	27/21	62.1 ± 6.1	59.8 ± 7.2	9	WHOCIST
Wang, 2020	OC	Chemotherapy and TCM	Chemotherapy	30 (F)	30 (F)	59.83 ± 8.82	56.30 ± 9.73	3	RECIST, QoL, KPS, and tumor marker
Liu, 2001	LC	Chemotherapy and TCM	Chemotherapy	28/12	15/5	54 ± 6.17	54 ± 5.89	7	Survival rate and KPS
Sun, 2012	MC	Chemotherapy and TCM	Chemotherapy	23/12	21/10	54.48 ± 13.11	60.06 ± 8.30	24	KPS, QoL, and tumor marker
Song, 2012	LC	Chemotherapy and TCM	Chemotherapy	13/7	9/11	58.80 ± 6.04	58.40 ± 5.52	8	RECIST, QoL, KPS, and tumor marker
Jiang, 2011	GC	Chemotherapy and TCM	Chemotherapy	23/12	21/10	60.85 ± 11.55	56.40 ± 7.84	8	RECIST, QoL, KPS, and tumor marker
Li, 2020	LC	Chemotherapy and TCM	Chemotherapy	23/21	24/20	62.91 ± 3.47	63.05 ± 3.51	8	Metastasis rate, recurrence rate, tumor marker, and immunologic function
Pu, 2013	HM	Radiotherapy, chemotherapy, and TCM	Radiotherapy and chemotherapy	18/12	18/12	53.5 ± 21.8	48 ± 26.98	16	Peripheral hemogram
Yang, 2016	LC	Chemotherapy and TCM	Chemotherapy	16/13	18/11	59.12 ± 3.28	60.77 ± 2.41	8	WHOCIST
Zhang, 2018	EC	Chemotherapy and TCM	Chemotherapy	25/13	29/11	60.55 ± 7.38	62.45 ± 5.89	6	RECIST and KPS
Bai, 2009	GC	Chemotherapy and TCM	Chemotherapy	19/5	19/4	56.29 ± 10.34	55.87 ± 8.54	8	QoL, KPS, and WHOCIST
Zha, 2019	GC	Targeted therapy and TCM	Targeted therapy	16/14	13/17	67.03 ± 8.72	67.07 ± 8.40	8	RECIST, QoL, and tumor marker
Chen, 2017	GC	Chemotherapy and TCM	Chemotherapy and TCM placebo	22/12	22/12	57.97 ± 8.18	59.4 ± 11.87	8	QoL and KPS
Zhang, 2018	GC	Chemotherapy and TCM	Chemotherapy	34/14	30/13	52.34 ± 4.44	53.65 ± 5.02	9	WHOCIST, KPS, immunologic function, and blood coagulation function
Wu, 2017	LC	TCM and BSC	Chemotherapy and BSC	22/8	19/11	58.73 ± 10.29	59.17 ± 8.32	8	RECIST, KPS, and blood coagulation function
Wang, 2008	CRC	Chemotherapy and TCM	Chemotherapy	20/14	22/12	52.58 ± 8.12	51.11 ± 7.72	12	WHOCIST
Tang, 2017	GC	Chemotherapy and TCM	Chemotherapy	17/15	21/10	61.84 ± 8.86	58.81 ± 8.92	6	RECIST, KPS, blood coagulation function, and QoL
Li, 2012	LC	Chemotherapy and TCM	Chemotherapy	26/12	19/11	67.37 ± 3.82	68.76 ± 3.37	8	RECIST, immunologic function, blood coagulation function, and median survival time
Zhang, 2013	MC	Chemotherapy and TCM	Chemotherapy	13/14	14/10	62.04 ± 12.08	61.3 ± 10.92	8	RECIST, immunologic function, and blood coagulation function
Zhou, 2016	LIC	Cyberknife and TCM	Cyberknife	28/2	30/0	51.4 ± 11.63	54.73 ± 9.06	12	RECIST and KPS
Zhang, 2020	HM	Expectant treatment and TCM	Expectant Treatment	11/9	11/9	54.35 ± 13.07	58.8 ± 10.95	12	Peripheral hemogram
Dong, 2014	LC	Chemotherapy and TCM	Chemotherapy	23/17	22/18	56.83 ± 13.38	57.6 ± 12.84	12	QoL and blood coagulation function
Wang, 2019	LIC	RTVSNSGR and TCM	RTVSNSGR	21/18	22/17	54.14 ± 6.71	54.56 ± 6.60	24	RECIST and tumor marker
Liang, 2017	LIC	TACE and TCM	TACE	24/5	23/7	48.69 ± 9.80	50.7 ± 11.29	12	WHOCIST and immunologic function
Li, 2012	LIC	TCM and BSC	BSC	25/5	24/6	51.63 ± 10.74	54.63 ± 9.72	12	WHOCIST
Zhang, 2011	GC	Chemotherapy and TCM	Chemotherapy	13/7	12/8	60.15 ± 13.97	63.9 ± 2.03	8	RECIST, tumor marker, QoL, and KPS
Zhong, 2014	LIC	Chemotherapy, surgery, and TCM	Chemotherapy and surgery	53/7	50/10	46.7 ± 10.4	48.1 ± 11.1	48	Survival rate, Metastasis rate, blood coagulation function, and Tumor marker
Chen, 2010	OC	Chemotherapy and TCM	Chemotherapy	20 (F)	20 (F)	55.20 ± 12.36	56.85 ± 9.07	8	RECIST and QoL
Xia, 2019	GC	Chemotherapy and TCM	Chemotherapy	19/11	23/7	63.53 ± 13.41	62.4 ± 11.24	6	RECIST, QoL, and blood coagulation function
Zhang, 2020	LC	Chemotherapy and TCM	Chemotherapy	23/7	21/9	63.97 ± 7.67	61.7 ± 9.83	6	RECIST, tumor marker, and KPS
Quan, 2018	GC	Chemotherapy and TCM	Chemotherapy	23/7	22/8	52.10 ± 8.71	49.2 ± 7.10	6	WHOCIST
Wang, 2018	BC	Endocrinotherapy and TCM	Endocrinotherapy	65 (F)	65 (F)	58.80 ± 7.04	51.85 ± 7.84	24	QoL, immunologic function, and tumor marker
Xu, 2010	PC	Chemotherapy and TCM	Chemotherapy	14/4	15/3	67.72 ± 6.27	69.33 ± 5.62	6	WHOCIST and blood coagulation function
Zhang, 2020	LC	Chemotherapy and TCM	Chemotherapy	16/14	17/13	67.43 ± 3.75	68.01 ± 4.02	6	WHOCIST
Guan, 2014	LC	Chemotherapy and TCM	Chemotherapy	18/12	21/9	59.70 ± 8.40	60.60 ± 7.80	12	RECIST and tumor marker
Li, 2011	CRC	Chemotherapy and TCM	Chemotherapy	12/8	10/10	61.3 ± 8.79	56.95 ± 13.90	8	RECIST, tumor marker, KPS, and QoL
Zhang, 2019	LC	Chemotherapy and TCM	Chemotherapy and placebo	8/22	6/24	61.93 ± 6.17	62.03 ± 6.47	24	RECIST and KPS
Wu, 2017	LC	Cyberknife treatment and TCM	Cyberknife treatment	41/19	43/17	56.13 ± 9.45	55.59 ± 10.03	12	WHOCIST, KPS, and tumor marker
Zhang, 2016	NSCLC	Chemotherapy and TCM	Chemotherapy	18/11	16/12	50.07 ± 13.24	47.76 ± 13.72	6	RECIST
Gao, 2018	LIC	Cyberknife treatment and TCM	Cyberknife treatment	23/7	22/8	55.03 ± 13.21	55.27 ± 12.89	8	WHOCIST, KPS, and tumor marker
Wei, 2018	LC	Cyberknife treatment and TCM	Cyberknife treatment	24/16	26/16	56.85 ± 7.36	57.28 ± 7.59	13	RECIST and KPS
Li, 2014	LC	Chemotherapy and TCM	Chemotherapy	22/8	24/6	63.95 ± 10.77	65.65 ± 10.60	8	RECIST, KPS, and blood coagulation function
Yao, 2018	MC	BSC and TCM	BSC	28/17	32/13	64.91 ± 6.80	65.33 ± 7.60	8	RECIST, KPS, and blood coagulation function
Yu, 2007	LIC	Chemotherapy and TCM	Chemotherapy	46/14	47/13	57.91 ± 9.12	57.76 ± 10.71	8	WHOCIST, tumor marker, and immunologic function
Wang, 2007	GC	Chemotherapy and TCM	Chemotherapy	22/1	21/2	62.17 ± 8.00	57.65 ± 9.94	6	WHOCIST and immunologic function
Li, 2017	LC	Chemotherapy and TCM	Chemotherapy	20/13	19/14	56.37 ± 10.42	57.03 ± 10.26	4	RECIST and immunologic function
Lu, 2012	LIC	Chemotherapy and TCM	Chemotherapy	11/9	8/12	44.00 ± 5.98	50 ± 6.34	48	WHOCIST and tumor marker
Hu, 2016	LIC	TACE and TCM	TACE	27/13	34/6	52.48 ± 12.02	51.48 ± 10.64	12	WHOCIST, tumor marker, KPS, and QoL
Zhong, 2016	LIC	TACE and TCM	TACE	59/21	68/12	45.25 ± 10.69	47.78 ± 12.30	24	WHOCIST, survival rate, and median survival time
Sun, 2014	CRC	Chemotherapy and TCM	Chemotherapy	16/7	14/8	61.00 ± 10.37	59.55 ± 8.95	8	RECIST, tumor marker, KPS, and QoL
Ye, 2004	GC	Chemotherapy and TCM	Chemotherapy	9/6	11/4	59.67 ± 13.69	60.00 ± 11.22	8	WHOCIST, tumor marker, and immunologic function
Duanmu, 2018	CRC	Chemotherapy and TCM	Chemotherapy	18/12	15/15	61.27 ± 11.77	62.13 ± 11.09	6	RECIST, tumor marker, KPS, and blood coagulation function
Xu, 2017	CRC	Chemotherapy and TCM	Chemotherapy	13/9	15/6	60.64 ± 10.05	62.67 ± 6.07	6	RECIST, tumor marker, and KPS
Shi, 2019	CRC	Chemotherapy and TCM	Chemotherapy	16/9	13/10	65.60 ± 8.45	63.17 ± 9.60	8	RECIST, tumor marker, KPS, and QoL
Yu, 2010	CRC	Chemotherapy and TCM	Chemotherapy	12/8	13/7	61.70 ± 7.63	60.60 ± 8.37	6	WHOCIST, tumor marker, KPS, and QoL
Xue, 2017	CRC	Chemotherapy and TCM	Chemotherapy	24/14	25/13	62.66 ± 8.91	61.18 ± 8.24	8	RECIST, KPS, and QoL
Shen, 2020	GC	Targeted therapy and TCM	Targeted therapy	20/3	18/4	67.13 ± 6.63	66.32 ± 6.24	4	RECIST, tumor marker, immunologic function, and KPS
Zhang, 2011	GC	Chemotherapy and TCM	Chemotherapy	16/4	14/6	58.25 ± 8.435	55.85 ± 13.74	8	RECIST, tumor marker, and QoL
Ding, 2007	CRC	Chemotherapy and TCM	Chemotherapy	9/11	11/9	58.00 ± 10.60	57.00 ± 10.19	8	WHOCIST
Li, 2017	LIC	Targeted therapy and TCM	Targeted therapy	21/9	23/7	67.43 ± 5.57	69.23 ± 7.05	12	RECIST, tumor marker, immunologic function, and KPS
Xu, 2012	GC	Chemotherapy and TCM	Chemotherapy	12/8	13/7	54.7 ± 11.67	58.7 ± 10.95	7	RECIST, tumor marker, QoL, and KPS
Dai, 2014	PC	Chemotherapy and TCM	Chemotherapy	14/11	13/12	55.20 ± 13.9	56.4 ± 14.80	12	WHOCIST, immunologic function, and blood coagulation function
Ran, 2019	LIC	TACE and TCM	TACE	30/11	28/13	48.35 ± 11.02	51.26 ± 12.07	4	WHOCIST, blood coagulation function, KPS, tumor marker
Yu, 2011	CRC	Chemotherapy and TCM	Chemotherapy	12/8	11/9	60.10 ± 9.40	59.95 ± 9.60	6	WHOCIST and QoL
Zhang, 2017	LIC	TACE and TCM	TACE	26/12	25/13	39.61 ± 5.42	39.42 ± 5.37	13	WHOCIST, immunologic function, and tumor marker

BC, breast cancer; BSC, best supportive care (cancer treatment); CRC, colorectal cancer; EC, esophagus cancer; GBC, gallbladder carcinoma; GC, gastric cancer; HM, hematological malignancies; KPS, Karnofsky score; LC, lung cancer; LIC, liver cancer; MC, multiple types of cancer; OC, ovarian cancer; PC, pancreatic cancer; QoL, quality of life; RECIST, Response Evaluation Criteria in Solid Tumors; RTVSNSGR, real-time virtual sonography navigation system guided radio frequency; TACE, transcatheter arterial chemoembolization; TCM, Traditional Chinese Medicine (TCM: *HuoxueHuayu* therapy); WHOCIST, World Health Organization Criteria in Solid Tumors.

**Table 3:** Quality assessment of the included studies.

Study	Selection bias		Performance bias		Attrition bias	Reporting bias	Other bias				Jadad score
	Random sequence	Allocation concealment	Blinding of participants	Blinding of outcome assessment	Incomplete outcome data	Selective reporting	Timing of outcome assessment	Similar at baseline	Compliance acceptable in all groups	Rationale for control group	
Qi, 2011	*H*	*U*	*U*	*U*	*L*	*L*	*L*	*L*	*L*	*L*	3
Zhou, 2020	*U*	*U*	*U*	*U*	*L*	*L*	*L*	*L*	*L*	*L*	4
Zhang, 2013	*L*	*U*	*U*	*U*	*L*	*L*	*L*	*L*	*L*	*L*	5
Huang, 2015	*L*	*L*	*U*	*U*	*L*	*L*	*L*	*L*	*L*	*L*	6
Zhou, 2013	*L*	*U*	*U*	*U*	*L*	*L*	*L*	*L*	*L*	*L*	5
Wang, 2020	*L*	*U*	*U*	*U*	*L*	*L*	*L*	*L*	*L*	*L*	5
Liu, 2001	*U*	*U*	*U*	*U*	*L*	*L*	*L*	*L*	*L*	*L*	4
Sun, 2012	*U*	*U*	*U*	*U*	*L*	*L*	*L*	*L*	*L*	*L*	4
Song, 2012	*L*	*U*	*U*	*U*	*L*	*L*	*L*	*L*	*L*	*L*	5
Jiang, 2011	*L*	*U*	*U*	*U*	*L*	*L*	*L*	*L*	*L*	*L*	5
Li, 2020	*L*	*U*	*U*	*U*	*L*	*L*	*L*	*L*	*L*	*L*	5
Pu, 2013	*L*	*U*	*U*	*U*	*L*	*L*	*L*	*L*	*L*	*L*	5
Yang, 2016	*U*	*U*	*U*	*U*	*L*	*L*	*L*	*L*	*L*	*L*	4
Zhang, 2018	*H*	*U*	*U*	*U*	*L*	*L*	*L*	*L*	*L*	*L*	3
Bai, 2009	*U*	*U*	*U*	*U*	*L*	*L*	*L*	*L*	*L*	*L*	4
Zha, 2019	*U*	*U*	*U*	*U*	*L*	*L*	*L*	*L*	*L*	*L*	4
Chen, 2017	*L*	*L*	*L*	*U*	*L*	*L*	*L*	*L*	*L*	*L*	7
Zhang, 2018	*H*	*U*	*U*	*U*	*L*	*L*	*L*	*L*	*L*	*L*	3
Wu, 2017	*U*	*U*	*U*	*U*	*L*	*L*	*L*	*L*	*L*	*L*	4
Wang, 2008	*U*	*U*	*U*	*U*	*L*	*L*	*L*	*L*	*L*	*L*	4
Tang, 2017	*U*	*U*	*U*	*U*	*L*	*L*	*L*	*L*	*L*	*L*	4
Li, 2012	*U*	*U*	*U*	*U*	*L*	*L*	*L*	*L*	*L*	*L*	4
Zhang, 2013	*L*	*U*	*U*	*U*	*L*	*L*	*L*	*L*	*L*	*L*	5
Zhou, 2016	*L*	*U*	*U*	*U*	*L*	*L*	*L*	*L*	*L*	*L*	5
Zhang, 2020	*L*	*U*	*U*	*U*	*L*	*L*	*L*	*L*	*L*	*L*	5
Dong, 2014	*L*	*U*	*U*	*U*	*L*	*L*	*L*	*L*	*L*	*L*	5
Wang, 2019	*L*	*U*	*U*	*U*	*L*	*L*	*L*	*L*	*L*	*L*	5
Liang, 2017	*H*	*U*	*U*	*U*	*L*	*L*	*L*	*L*	*L*	*L*	3
Li, 2012	*U*	*U*	*U*	*U*	*L*	*L*	*L*	*L*	*L*	*L*	4
Zhang, 2011	*U*	*U*	*U*	*U*	*L*	*L*	*L*	*L*	*L*	*L*	4
Zhong, 2014	*U*	*L*	*U*	*U*	*L*	*L*	*L*	*L*	*L*	*L*	5
Chen, 2010	*U*	*U*	*U*	*U*	*L*	*L*	*L*	*L*	*L*	*L*	4
Xia, 2019	*L*	*U*	*U*	*U*	*L*	*L*	*L*	*L*	*L*	*L*	5
Zhang, 2020	*L*	*U*	*U*	*U*	*L*	*L*	*L*	*L*	*L*	*L*	5
Quan, 2018	*U*	*U*	*U*	*U*	*L*	*L*	*L*	*L*	*L*	*L*	4
Wang, 2018	*U*	*U*	*U*	*U*	*L*	*L*	*L*	*L*	*L*	*L*	4
Xu, 2010	*U*	*U*	*U*	*U*	*L*	*L*	*L*	*L*	*L*	*L*	4
Zhang, 2020	*L*	*U*	*U*	*U*	*U*	*L*	*L*	*L*	*L*	*L*	5
Guan, 2014	*L*	*U*	*U*	*U*	*L*	*L*	*L*	*L*	*L*	*L*	5
Li, 2011	*L*	*U*	*U*	*U*	*L*	*L*	*L*	*L*	*L*	*L*	5
Zhang, 2019	*L*	*L*	*L*	*U*	*L*	*L*	*L*	*L*	*L*	*L*	7
Wu, 2017	*H*	*U*	*U*	*U*	*L*	*L*	*L*	*L*	*L*	*L*	3
Zhang, 2016	*L*	*U*	*U*	*U*	*L*	*L*	*L*	*L*	*L*	*L*	5
Gao, 2018	*L*	*U*	*U*	*U*	*L*	*L*	*L*	*L*	*L*	*L*	5
Wei, 2018	*L*	*U*	*U*	*U*	*L*	*L*	*L*	*L*	*L*	*L*	5
Li, 2014	*U*	*U*	*U*	*U*	*L*	*L*	*L*	*L*	*L*	*L*	4
Yao, 2018	*L*	*U*	*U*	*U*	*L*	*L*	*L*	*L*	*L*	*L*	5
Yu, 2007	*L*	*U*	*U*	*U*	*L*	*L*	*L*	*L*	*L*	*L*	5
Wang, 2007	*U*	*U*	*U*	*U*	*L*	*L*	*L*	*L*	*L*	*L*	4
Li, 2017	*L*	*U*	*U*	*U*	*L*	*L*	*L*	*L*	*L*	*L*	5
Lu, 2012	*L*	*U*	*U*	*U*	*L*	*L*	*L*	*L*	*L*	*L*	5
Hu, 2016	*U*	*U*	*U*	*U*	*L*	*L*	*L*	*L*	*L*	*L*	4
Zhong, 2016	*L*	*U*	*U*	*U*	*L*	*L*	*L*	*L*	*L*	*L*	5
Sun, 2014	*L*	*U*	*U*	*U*	*L*	*L*	*L*	*L*	*L*	*L*	5
Ye, 2004	*U*	*U*	*U*	*U*	*L*	*L*	*L*	*L*	*L*	*L*	4
Duanmu, 2018	*L*	*U*	*U*	*U*	*L*	*L*	*L*	*L*	*L*	*L*	5
Xu, 2017	*U*	*U*	*U*	*U*	*L*	*L*	*L*	*L*	*L*	*L*	4
Shi, 2019	*L*	*U*	*U*	*U*	*L*	*L*	*L*	*L*	*L*	*L*	5
Yu, 2010	*U*	*U*	*U*	*U*	*L*	*L*	*L*	*L*	*L*	*L*	4
Xue, 2017	*U*	*U*	*U*	*U*	*L*	*L*	*L*	*L*	*L*	*L*	4
Shen, 2020	*U*	*U*	*U*	*U*	*L*	*L*	*L*	*L*	*L*	*L*	4
Zhang, 2011	*U*	*U*	*U*	*U*	*L*	*L*	*L*	*L*	*L*	*L*	4
Ding, 2007	*U*	*U*	*U*	*U*	*L*	*L*	*L*	*L*	*L*	*L*	4
Li, 2017	*U*	*U*	*U*	*U*	*L*	*L*	*L*	*L*	*L*	*L*	4
Xu, 2012	*L*	*U*	*U*	*U*	*L*	*L*	*L*	*L*	*L*	*L*	5
Dai, 2014	*L*	*U*	*U*	*U*	*L*	*L*	*L*	*L*	*L*	*L*	5
Ran, 2019	*L*	*U*	*U*	*U*	*L*	*L*	*L*	*L*	*L*	*L*	5
Yu, 2011	*U*	*U*	*U*	*U*	*L*	*L*	*L*	*L*	*L*	*L*	4
Zhang, 2017	*U*	*U*	*U*	*U*	*L*	*L*	*L*	*L*	*L*	*L*	4

**Table 4:** Summary of findings.

Outcomes	No. of participants (studies)	Certainty of the evidence (GRADE)	Relative effect (95% CI)	Anticipated absolute effects	
				Risk with conventional treatment	Risk difference with *HuoxueHuayu* therapy
RECIST	1977	⊕⊕⊕⊕	RR 1.44	258 per 1,000	113 more per 1,000
	(33 RCTs)	High	(1.27 to 1.63)		(70 more to 162 more)
WHOCIST	1721	⊕⊕⊕⊕	RR 1.40	279 per 1,000	112 more per 1,000
	(26 RCTs)	High	(1.23 to 1.59)		(64 more to 165 more)
Recurrence rate	208	⊕⊕◯◯	RR 0.85	567 per 1,000	85 fewer per 1,000
	(2 RCTs)	Low^a,b^	(0.72 to 0.99)		(159 fewer to 6 fewer)
KPS	1604	⊕⊕⊕◯	—	The mean KPS was 0	MD 5.6 higher
	(28 RCTs)	Moderate^c^			(5.04 higher to 6.15 higher)
APTT	479	⊕◯◯◯	—	The mean APTT was 0	MD 0.13 higher
	(7 RCTs)	Very low^b,c,d,e,f,g^			(2.29 lower to 2.54 higher)
PT	599	⊕◯◯◯	—	The mean PT was 0	MD 0.72 higher
	(8 RCTs)	Very low^b,c,d,e,f,g^			(0.65 lower to 2.1 higher)
CD4	798	⊕◯◯◯	—	—	SMD 1.25 higher
	(14 RCTs)	Very low^b,c,d,e,f^			(0.77 higher to 1.74 higher)
CD8	614	⊕◯◯◯	—	—	SMD 0.02 lower
	(11 RCTs)	Very low^b,c,d,e,f,g^			(0.51 lower to 0.47 higher)
CD4/CD8	809	⊕⊕◯◯	—	—	SMD 1.05 higher
	(13 RCTs)	Low^c,d,e,f^			(0.69 higher to 1.42 higher)
CD3	648	⊕⊕◯◯	—	—	SMD 1.23 higher
	(12 RCTs)	Low^c,d,e,f^			(0.79 higher to 1.66 higher)
NK	512	⊕◯◯◯	—	—	SMD 0.74 higher
	(8 RCTs)	Very low^b,e,f^			(0.32 higher to 1.15 higher)

^*∗*^The risk in the intervention group (and its 95% confidence interval) is based on the assumed risk in the comparison group and the relative effect of the intervention (and its 95% CI). CI: confidence interval; RR: risk ratio; MD: mean difference; SMD: standardized mean difference.					

GRADE working group grades of evidence. High certainty: we are very confident that the true effect lies close to that of the estimate of the effect. Moderate certainty: we are moderately confident in the effect estimate: the true effect is likely to be close to the estimate of the effect, but there is a possibility that it is substantially different. Low certainty: our confidence in the effect estimate is limited: the true effect may be substantially different from the estimate of the effect. Very low certainty: we have very little confidence in the effect estimate: the true effect is likely to be substantially different from the estimate of effect.					

a: small sample size, b: publication bias, c: the statistical test for heterogeneity shows a low *P* value, d: point estimates vary widely across studies, e: confidence intervals (CIs) show minimal overlap, f: wide confidence intervals, and g: uncertainty about magnitude of effect.

## Data Availability

All data and materials generated and analyzed during the present study are available from the corresponding author upon reasonable request.

## Abbreviations

APTT: Activated partial thromboplastin time
BC: Breast cancer
BSC: Best supportive care (cancer treatment)
CRC: Colorectal cancer
D-D: D-dimer
EC: Esophagus cancer
FDP: Fibrinogen degradation products
FIB: Fibrinogen
GBC: Gallbladder carcinoma
GC: Gastric cancer
HM: Hematological malignancies
KPS: Karnofsky score
LC: Lung cancer
LIC: Liver cancer
LYM: Lymphocyte
MC: Multiple types of cancer
NK: Natural killer cell
OC: Ovarian cancer
PC: Pancreatic cancer
PLT: Platelets
PT: Prothrombin time
QoL: Quality of life
RBC: Red blood cell
RCT: Randomized controlled trial
RECIST: Response evaluation criteria in solid tumors
RTVSNSGR: Real-time virtual sonography navigation system guided radiofrequency
TACE: Transcatheter arterial chemoembolization
TCM: Traditional Chinese Medicine
WBC: White blood cell
WHOCIST: World Health Organization Criteria in Solid Tumors.
